# High-contrast photoacoustic imaging through scattering media using correlation detection of adaptive time window

**DOI:** 10.1038/s41598-019-53990-w

**Published:** 2019-11-21

**Authors:** Liqi Yu, Jialin Sun, Xinjing Lv, Qi Feng, Huimei He, Bin Zhang, Yingchun Ding, Qiang Liu

**Affiliations:** 1 College of Mathematics and Physics, Beijing University of Chemical Technology, Beijing, 100029 China; 2 State Key Laboratory of Precision Measurement Technology and Instruments, Department of Precision Instruments, Tsinghua University, Beijing, 100084 China

**Keywords:** Imaging and sensing, Microscopy

## Abstract

Photoacoustic imaging has the advantages of high contrast and deep imaging depth. However, with the increasing of imaging depth, the signal-to-noise ratio (SNR) of the detected signal decreases, due to the light scattering that seriously affects the recovery image quality. In this paper, we experimentally demonstrated that higher contrast photoacoustic imaging was achieved using photoacoustic wavefront shaping technology in the presence of light scattering and low SNR signals. The imaging contrast is improved from 1.51 to 5.30. More importantly, we propose a dynamic time window method for the photoacoustic signal extraction algorithm, named correlation detection of adaptive time window, which further improves the contrast of photoacoustic imaging to 9.57. Our method effectively improves the contrast of photoacoustic imaging through scattering media.

## Introduction

Photoacoustic imaging technology is one of the research focuses in microscopic imaging. Biological tissue absorbs pulse laser to excite thermal expansion to produce an outwardly radiated ultrasonic signal, a phenomenon known as photoacoustic effect^1^. Photoacoustic imaging utilizes ultrasonic signals in photoacoustic effect for imaging. Common optical imaging methods are affected by scattering property of biological tissue, and have shallow imaging depth. Different from optical imaging methods, the scattering of ultrasound is two to three orders of magnitude weaker than the light scattering in biological tissue^2^, so the penetration depth of photoacoustic imaging is deeper than that of optical imaging methods. Besides, photoacoustic imaging also has the advantage of high contrast^1^. In recent years, photoacoustic imaging technology has been widely applied in the field of biomedicine. It provides very promising application in the research of blood vessels testing, cancer detection and so on^3–5^. However, in the deeper region of the tissue, the incident light (the pulse laser utilized to excite the photoacoustic signal) is seriously scattered. And with the increasing of imaging depth, the light scattering becomes more severe. This scattering seriously reduces the energy of the pulse laser utilized to excite the photoacoustic signal, resulting in a low signal-to-noise ratio (SNR) of the photoacoustic signal. Traditional photoacoustic imaging methods rarely consider the influence of scattering of the incident light^6,7^.

The emergence of wavefront shaping technology makes it possible to use diffuse light for focusing and imaging, which can actually improve the imaging depth^8^. By modulating the amplitude and phase of the incident light before it arrives at scattering media, an optical focus can be formed behind the scattering media. Photoacoustic wavefront shaping technology combining photoacoustic effect and wavefront shaping uses the ultrasonic signal generated by photoacoustic effect as a noninvasive feedback signal to modulate the incident light, and can achieve high contrast imaging and improve imaging depth. Caravaca-Aguirre A. M. *et al*. combined photoacoustic imaging and wavefront shaping to effectively improve the spatial resolution of imaging^9^. Conkey D. B. *et al*. further improved the resolution of the photoacoustic wavefront shaping imaging by using the spatially non-uniform sensitivity of the focused ultrasound transducer to the focus region^10^. However, most of the wavefront modulate devices used in the study are liquid crystal spatial light modulators (SLMs)^9,10^. And it is difficult to apply in living tissue with short decorrelation time due to the limitation of the low refresh rate of SLM (~100 Hz). Digital micromirror device (DMD) whose refresh rate is 22 kHz can greatly improve the speed of optimization. Yet compared to SLM (<40 mJ/cm^2^), the light intensity threshold of DMD surface is lower (<200 μJ/cm^2^). As a result, the energy of the incident light used for exciting the ultrasound is lower, which leads to a lower SNR of the photoacoustic signal^11^. In wavefront shaping process, the wavefront of the incident light are optimized, and the light intensity of the focus in the biological tissue is improved, so the amplitude of the photoacoustic signal gets enhancement. The essence of imaging based on photoacoustic signals captured by ultrasound transducers is the procedure of digital signal processing. In previous studies, the peak-to-peak value of the photoacoustic signal was directly used as a reference for the optical focus optimization during imaging. Even so, the clutters generated by devices’ electrostatic and amplifier noise still have a great influence on the imaging contrast of final recovered image.

In this paper, we experimentally demonstrated that higher contrast photoacoustic imaging was achieved using photoacoustic wavefront shaping technology in the presence of light scattering and low SNR signals. The imaging contrast is improved from 1.51 to 5.30. More importantly, we propose a dynamic time window method for the photoacoustic signal extraction algorithm, named correlation detection of adaptive time window. Our method eliminates the interference of clutters and noise. Besides, our method eliminates the time deviation of photoacoustic signals from different positions to ultrasound transducer. The contrast of photoacoustic imaging is further improved to 9.57. Our method effectively improves the contrast of photoacoustic imaging through scattering media.

## Experimental Setup

**Figure 1:**
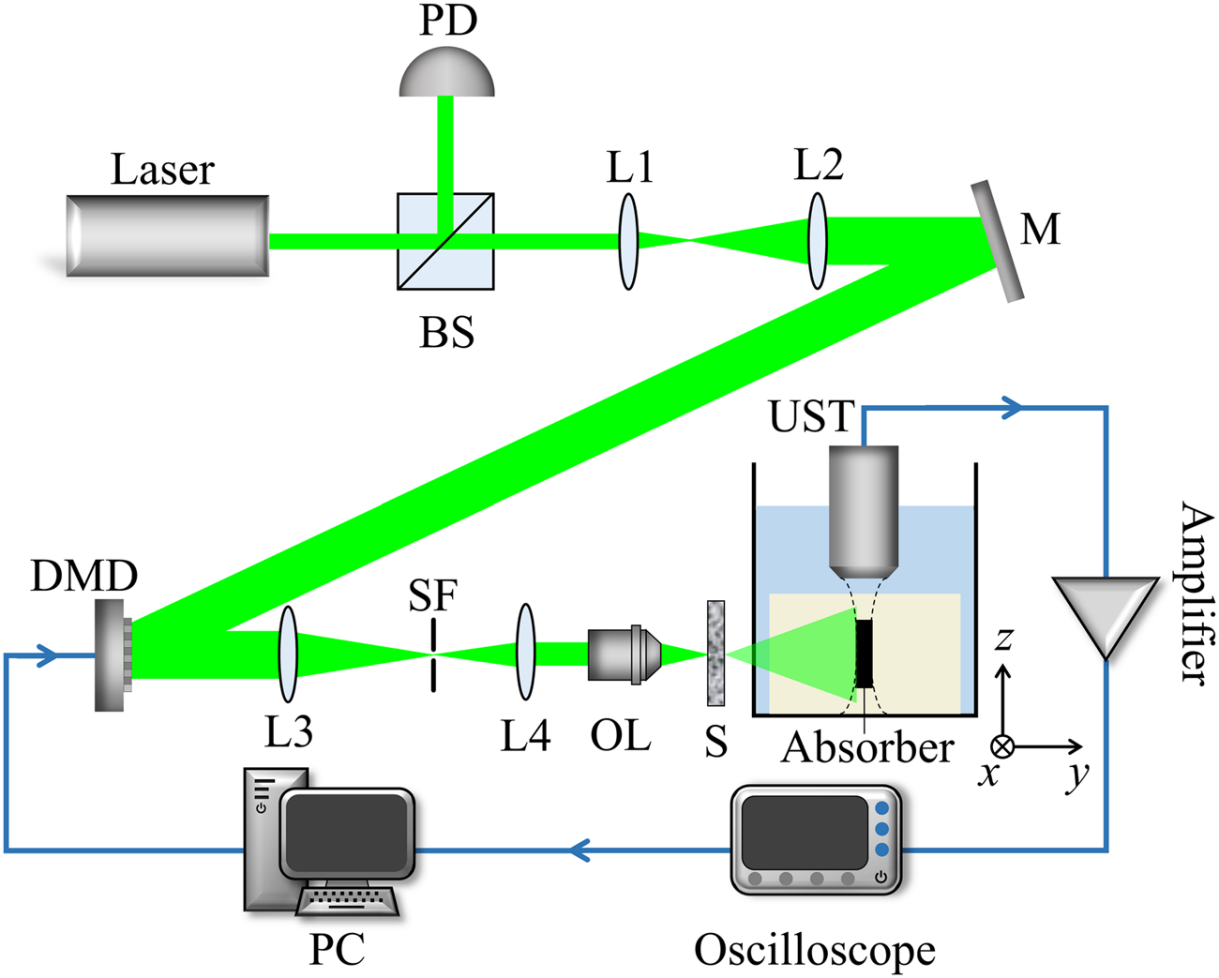
Experimental configuration. BS: Beam splitter; PD: Photodiode; L1, L2, L3, L4: Lens; M: Mirror; SF: Spatial filter; OL: Objective lens; S: Scattering diffuser; UST: Ultrasound transducer; The absorber is embedded in an agarose gel and placed in a water tank. The absorber is perpendicular to both the laser irradiation direction and the axial direction of UST. The blue line denotes the circuit part in the experiment.

The experimental configuration is shown in Fig. 1. The optical pluses emitted from a commercial pulse laser source (LABest, SGR-10) have a 532 nm wavelength with a repetition rate of 10 Hz and a pulse energy of ~800 μJ. The laser beam was expanded and collimated, then it was reflected by the mirror onto the DMD (Texas Instruments, DLP6500). The light reflected by the DMD was constricted and filtered by a 4 *f* system and then was focused by the objective lens (10 × , NA = 0.25) onto the scattering diffuser (Edmund, Diffuser 83419). A portion of the beam was split into the photodiode (Hamamatsu Photonics, S5971) to compensate for the energy fluctuations of the pulse laser. The photoacoustic signals were generated from an absorber embedded in an agarose gel. The absorber used in our experiment was 100 μm diameter black nylon thread, which was placed 2.5 cm behind the scattering diffuser to produce speckles approximate to the absorber. Similarly, in order to improve the optimization results, the speckle size set in the experiment matched the size of the absorber. The resolution of the imaging system was also determined by the speckle size^9^. A 20 MHz ultrasound transducer (Olympus, V317) was used to collect the photoacoustic signals. The photoacoustic signals were amplified by the amplifier (Mini-Circuits, ZFL-500LN + ) and then acquired by the oscilloscope (LeCroy, 806Zi-A). The computer dealt with the data digitized by the oscilloscope in real-time and refreshed the mask of the DMD according to the photoacoustic feedback signal. The water tank was placed on a two-dimensional translation stage (Daheng, GCD-203050M/301101 M) and moved in the *x-z* plane.

**Figure 2:**
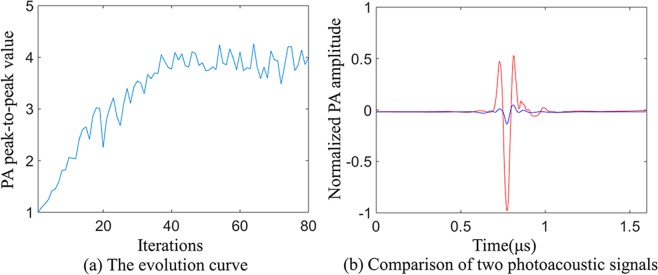
Genetic algorithm optimization process. (**a**) is the evolution curve of the genetic algorithm, and (**b**) is the comparison of the photoacoustic signals before and after optimization. The blue line and the red line represent the photoacoustic signal before and after wavefront shaping optimization, respectively.

In the wavefront shaping process, the incident light was optimized by genetic algorithm to enhance the amplitude of the photoacoustic signals. Genetic algorithm used photoacoustic signals as feedback signals^12^. Random masks were generated as the initial populations, which were ranked in descending order by the fitness of the initial populations. In the experiment, the fitness value was the peak-to-peak value of the photoacoustic signal acquired by the oscilloscope. Masks with high fitness values in each generation were easier to be selected by PC to generate next generation by crossing and mutating, and were loaded on the DMD. Through multiple iterations, an optimal mask, i.e., the mask with the largest peak-to-peak value of photoacoustic signal at the target point, was obtained^13^. In the experiment, we set 80 generations, and each generation contains 50 populations. The result of wavefront shaping optimization is shown in Fig. 2, in which the peak-to-peak value of photoacoustic signal reaches about 4 through 80 iterations and the photoacoustic signal is enhanced by about 8.05 times. After wavefront shaping optimization, DMD constantly displayed the optimal mask during scanning and imaging.

## Scanning and Imaging

After the enhancement of the photoacoustic signal by wavefront shaping optimization, we experimentally demonstrated two-dimensional scanning and imaging. We kept the scattering diffuser and the ultrasound transducer at a fixed position. The absorber and the water tank moved with the *x-z* two-dimensional translation stage.

**Figure 3:**
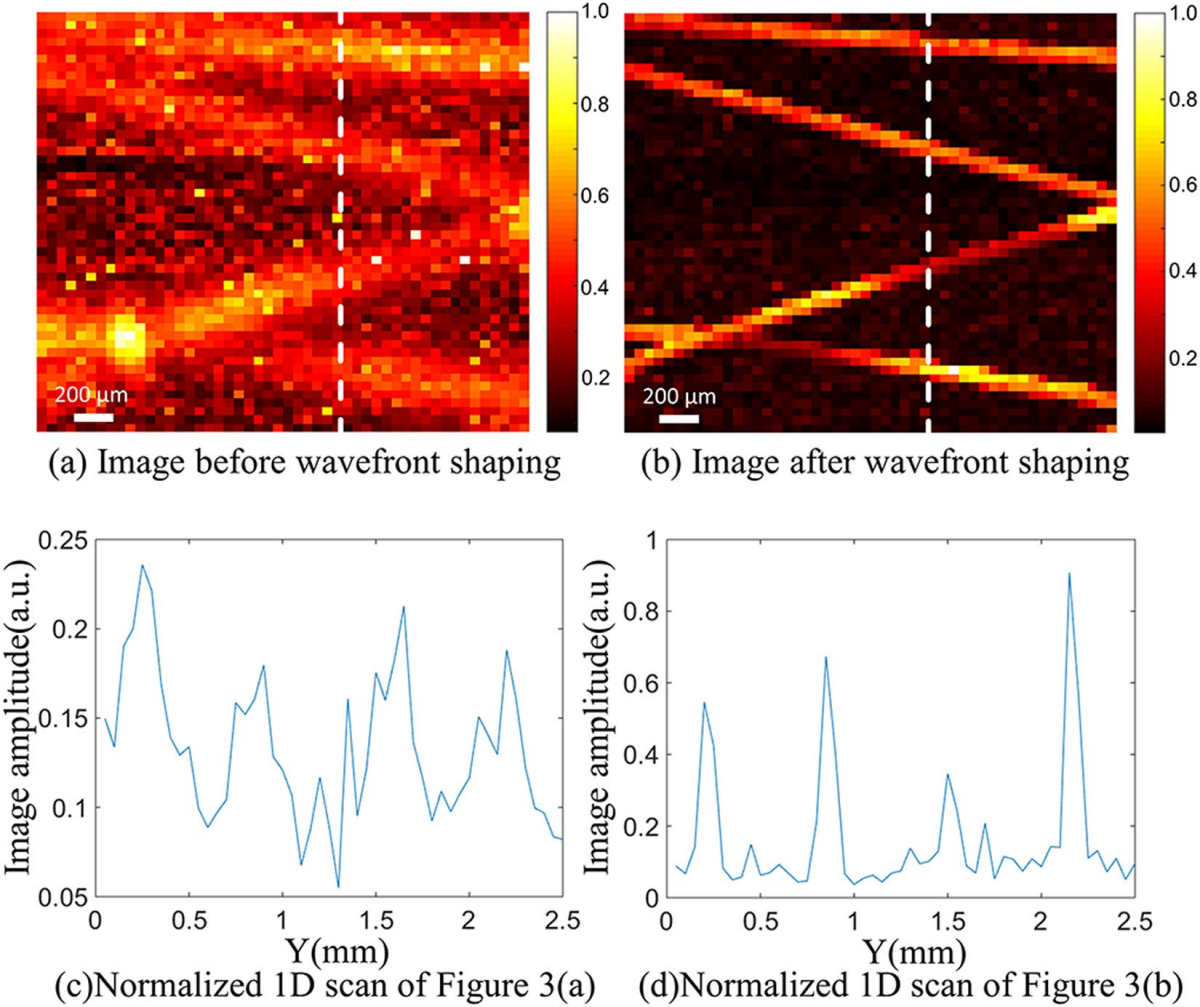
2D Maximum intensity projection images. (**a**) is a scanned image before wavefront shaping, and (**b**) is a scanned image after wavefront shaping. (**c,d**) are normalized 1D scans of white dashed lines in (**a,b**).

**Figure 4:**
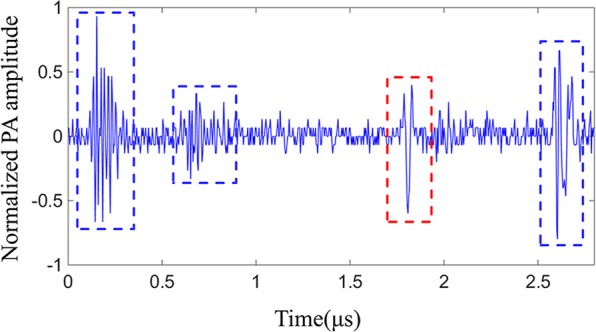
Photoacoustic signal acquired in the experiment. The signal in the red block is the photoacoustic signal, and the signals in the blue blocks are the clutters.

We scanned four crossed nylon threads before and after wavefront shaping optimization respectively. The photoacoustic signal amplitude, recorded from each position (*x-z* plane), was processed to reconstruct the 2D maximum intensity projection of the four crossed nylon threads. The imaging results are shown in Fig. 3(a,b). In order to evaluate the contrast of the image, one can use a contrast calculation formula, known as the Weber contrast, defined as^14^:

1 $$W=\frac{I-{I}_{b}}{{I}_{b}}$$

where *I* and *I*_*b*_ represent the luminance of the image and the background, respectively. The contrast of the image before wavefront shaping is 1.51, and the contrast of the image after wavefront shaping is 5.30. Since the energy of the speckle field is relatively dispersed before wavefront shaping, the image of the pre-optimized image has a poor imaging resolution and low contrast. After wavefront shaping optimization, the scattered light is focused on the absorber, and the resolution and contrast of the image are improved effectively. However, due to the presence of clutters and Gaussian white noise, the contrast of image is not satisfactory. The normalized 1D scans at the *x* = 1.60 mm profile (white dashed line) of Fig. 3(a,b) are shown in Fig. 3(c,d). From the 1D scans, we can also see that the contrasts of the images are poor due to the interference of clutters and Gaussian white noise. The clutters were caused by the devices’ electrostatic and amplifier noise during the experiment. Figure 4 shows a photoacoustic signal mixed with clutters acquired by the oscilloscope. The signal in the red block is the photoacoustic signal, and the signals in the blue blocks are the clutters. Clutters appear randomly in the time domain. When clutters and photoacoustic signal overlap in the time domain, the waveform and the amplitude of the photoacoustic signal will be seriously affected.

**Figure 5:**
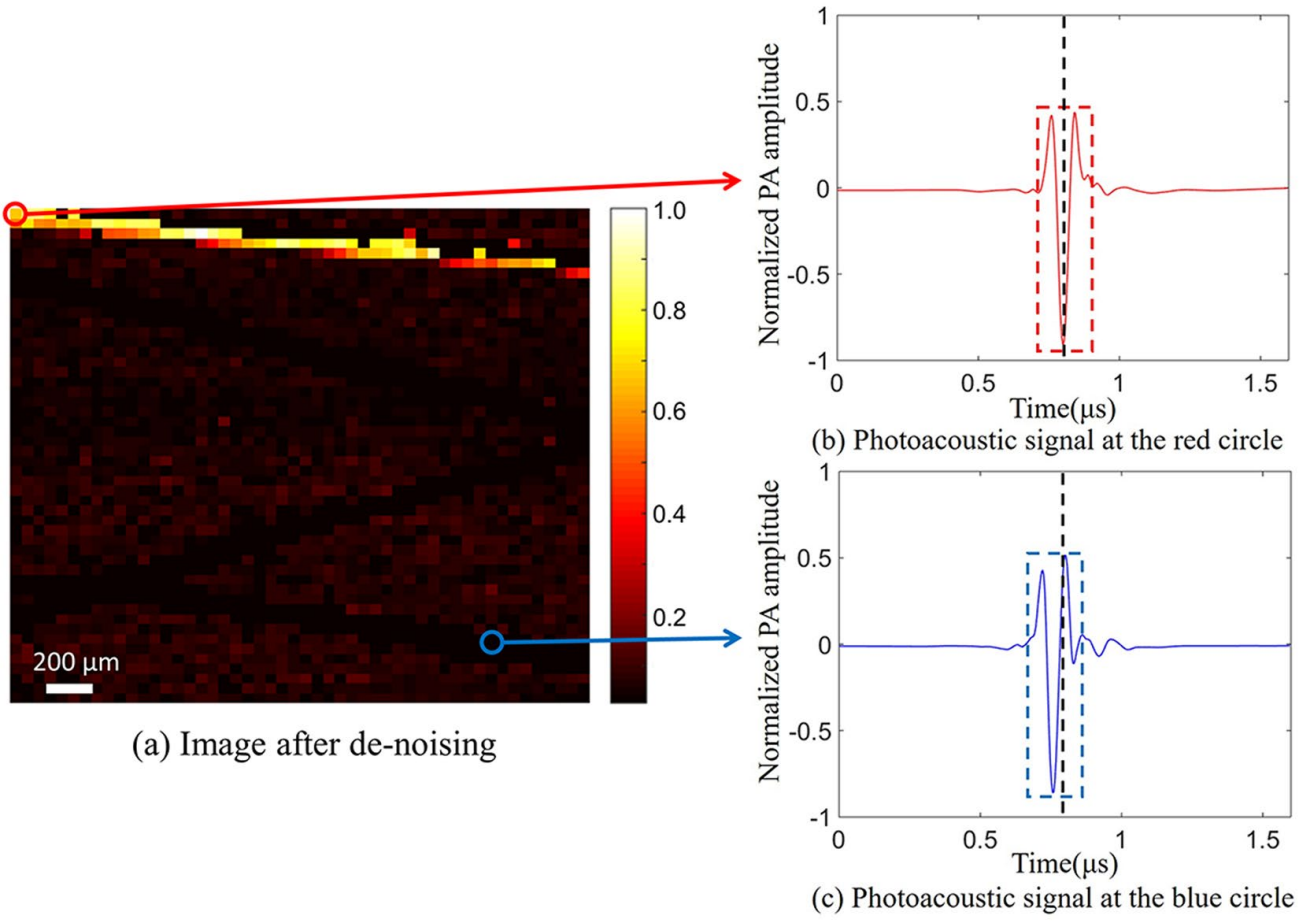
2D maximum intensity projection image. (**a**) is the recovered photoacoustic image applying the photoacoustic signal extraction algorithm. (**b**) is the photoacoustic signal at the red circle (the first point). (**c**) is the photoacoustic signal at the blue circle (the 2340th point).

In order to eliminate the interference of clutters and noise and improve the imaging contrast, we applied a photoacoustic signal extraction algorithm based on wavelet de-noising and correlation detection in the scanning and imaging process. Details about the photoacoustic signal extraction algorithm are given in our previous paper^15^. Firstly, the signal was de-noised by wavelet de-noising to remove Gaussian white noise. The mother wavelet was a fourth-order daubechies wavelet similar to our photoacoustic signal waveform^16^. Then, the correlation detection was used to calculate the correlation coefficient between each acquired signal and the template signal. The normalized correlation coefficient is calculated as equation^17^:

2 $${\gamma }_{xy}=\frac{{\sum }_{n=0}^{N-1}[x(n)-\bar{x}][y(n)-\bar{y}]}{\sqrt{{\sum }_{n=0}^{N-1}{[x(n)-\bar{x}]}^{2}{\sum }_{n=0}^{N-1}{[y(n)-\bar{y}]}^{2}}}$$

where *γ*_*xy*_ is the normalized correlation coefficient, *N* is the number of points of the template signal, *x*(*n*) are the template signal points, *y*(*n*) are the signal points under analysis, $\bar{x}$ is the mean of the template signal points, and $\bar{y}$ is the mean of signal points. The value of the normalized correlation coefficient is between −1 and 1, and 1 denotes the exact match between the signal under analysis and the template signal. In the experiment, the signal whose correlation coefficient was lower than the threshold value, that is, the photoacoustic signal was interfered by clutters, was filtered out. The photoacoustic signals of each position were collected five times, and the average values of the peak-to-peak value of these signals were calculated as the maximum intensity projection of the image. The template signal used for correlation detection was the photoacoustic signal at the first point of the image and the threshold value of the correlation coefficient was 0.7. The imaging result is shownin Fig. 5(a), which is a projection image of the maximum intensity of the photoacoustic signal at each position. In scanning and imaging, we obtained photoacoustic signals at each position and calculated their peak-to-peak values (the maximum value of the signal minus the minimum value) as the maximum intensity of their photoacoustic signals, then normalized the maximum intensities of these signals as a recovered photoacoustic image. We find that wavelet de-noising and correlation detection can effectively eliminate clutters and Gaussian white noise. However, after applying the photoacoustic signal extraction algorithm, at the position where photoacoustic signal should be extracted by the photoacoustic signal extraction algorithm, the photoacoustic signal cannot be recognized. It should be noted that the extraction of photoacoustic signals in Fig. 3(a,b) did not apply wavelet de-noising and correlation detection, so this problem did not occur.

The photoacoustic signal waveforms at the first point (red circle) and at the 2340th point (blue circle) of Fig. 5(a) are shown in Fig. 5(b,c), respectively. We can see that the relative time of these two signals has a deviation in time domain (black dashed lines in Fig. 5(b,c)). In fact, the ultrasound transducer used in the experiment has a focal region of 200 μm and the nylon thread has a diameter of 100 μm. Therefore, during scanning and imaging, the change of the relative position of the absorber and the ultrasound transducer causes the change of the time of the photoacoustic signal transmitting to the ultrasound transducer. Although the photoacoustic signals at the two positions are similar, the correlation coefficient between these two signals is very low (−0.6719, Fig. 5(b,c)). This is mainly due to the different time the transducer receives the signal. Eventually, at the position where photoacoustic signal should be extracted, the photoacoustic signal cannot be recognized.

**Figure 6:**
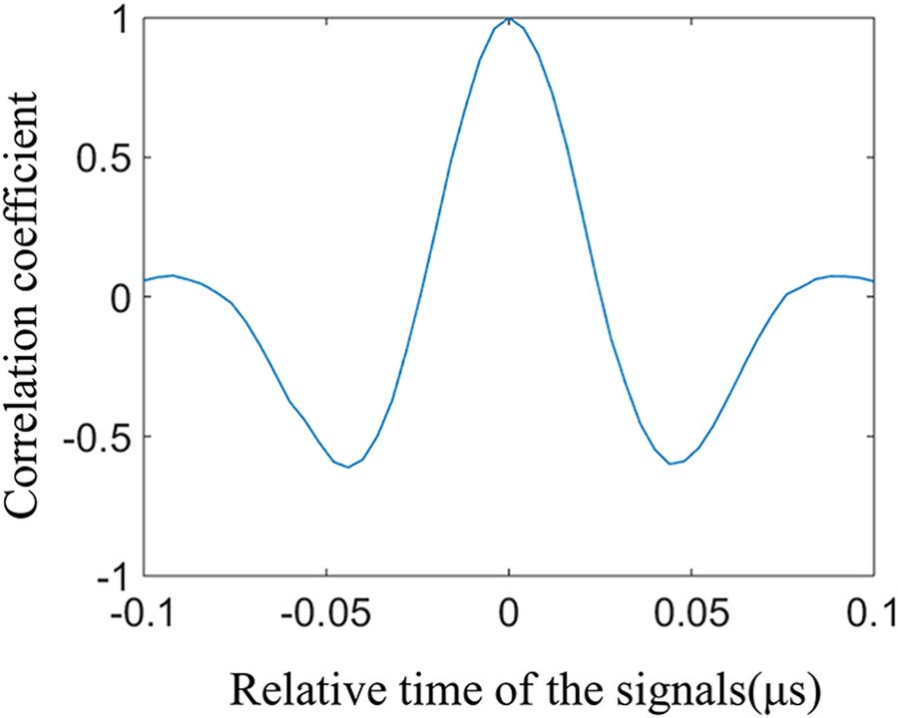
The relationship between the correlation coefficient of the photoacoustic signal and template signal versus their relative time.

Figure 6 shows the relationship between the correlation coefficient of the photoacoustic signal and template signal versus their relative time, in which a positive relative time value indicates that the signal is acquired after the template and a negative relative time value indicates that the signal is acquired before the template. From the figure we can see that, with the changing of relative time, the correlation coefficient of the photoacoustic signal and the template signal fluctuates between −0.7 and 1. The negative value of the correlation coefficient is mainly caused by the fact that the crest of the photoacoustic signal overlaps with the trough of the template signal. In our experiments, different waveforms of the photoacoustic signals and the noises were collected, and their correlation coefficients were calculated. The correlation coefficients for the photoacoustic signals corresponding to different masks are above 0.8, and the correlation coefficients between photoacoustic signals and noises are below 0.4. Therefore, we set the correlation coefficient threshold to 0.7. If the correlation coefficient of template signal and photoacoustic signal is lower than the threshold value, the photoacoustic signal extraction algorithm will recognize it as a clutter and filter it out.

To solve this problem, we propose a dynamic time window method for the photoacoustic signal extraction algorithm, named correlation detection of adaptive time window. The method dynamically finds the lowest point of the photoacoustic signal, which is used as the reference point to select the time window. Thus, the influence on the correlation coefficient caused by the difference in relative time of the photoacoustic signal and template signal is eliminated.

**Figure 7:**
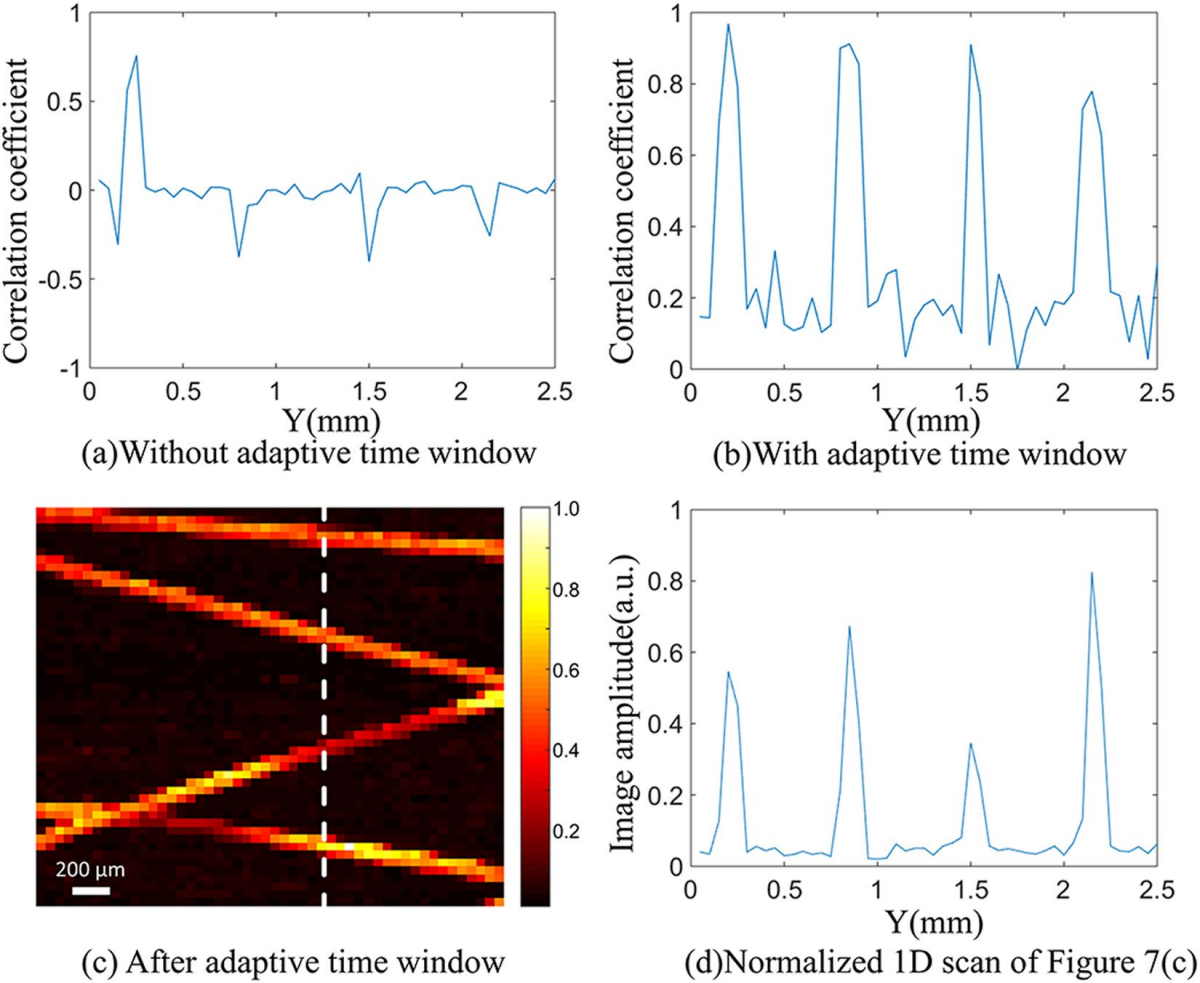
Figures of using correlation detection of adaptive time window. (**a,b**) indicate the correlation coefficient without using adaptive time window and with using adaptive time window (the white dashed line of Fig. 3(b)) respectively. (**c**) is the final recovered image of the extracted photoacoustic signals using correlation detection of adaptive time window. (**d**) is the normalized 1D scan of white dashed lines in (**c**).

The correlation coefficient between the photoacoustic signal and the template signal at the corresponding white dashed line in Fig. 3(b) is shown in Fig. 7(a). Figure 7(b) shows the values of the correlation coefficients using correlation detection of adaptive time window. Through the adaptive window method, the correlation coefficient between the photoacoustic signal and the template signal is significantly improved. And signals can be effectively distinguished from the clutters by setting an appropriate threshold value. Figure 7(c) shows the final recovered image of the extracted photoacoustic signals using correlation detection of adaptive time window. The contrast of Fig. 7(c) is calculated to be 9.57. Due to the application of correlation detection of adaptive time window, clutters and noise are well filtered out. Figure 7(d) is the 1D scan of the image at the white dashed line position *x* = 1.60 mm, which further demonstrates the improvement of imaging contrast.

## Discussion and Conclusion

In this experiment, the wavefront shaping process takes about 30 minutes, and the scanning and imaging process takes about 1 hour. Applying wavelet de-noising and correlation detection of adaptive time window, we do not need to spend more time to acquire a large number of photoacoustic signals for averaging to improve the SNR. Currently, the speed of focusing and imaging is mainly limited by the low pulse laser repetition rate. With a higher repetition rate laser source and a faster calculating device (e.g. FPGA), sub-second optimization of the incident light may be implemented^18^.

We have experimentally demonstrated the ability to focus light through scattering media using a faster wavefront modulation device (DMD). We obtained the optimal DMD mask by wavefront shaping. The amplitude of the photoacoustic signal is enhanced by about 8.05 times compared to using a random mask. Different from SLM, DMD can only modulate the amplitude of incident light, which leads to a lower enhancement of photoacoustic signals after wavefront shaping, but its faster refresh rate reduces the experimental time. We further demonstrated the ability to scan and image using enhanced photoacoustic signals. The pulse laser is seriously scattered before the wavefront shaping, so the resolution and contrast of the image are poor. After wavefront shaping, the energy of the speckle field is concentrated, thereby improving the resolution and contrast of the image. After wavefront shaping, we improved the contrast of photoacoustic imaging from 1.51 to 5.30.

Furthermore, we applied the photoacoustic signal extraction algorithm based on wavelet de-noising and correlation detection to photoacoustic imaging. In complex electromagnetic environment, clutters and noise exist everywhere and are severe. Our proposed focusing and imaging methods can effectively eliminate the interference of clutters and noise. Considering the situation that the change of the relative position of the absorber and the ultrasound transducer causes the change of the time of the photoacoustic signal transmitting to the ultrasound transducer, we propose a dynamic time window method for the photoacoustic signal extraction algorithm, named correlation detection of adaptive time window. Our method eliminates the time deviation of photoacoustic signals from different positions to ultrasound transducer. Correlation detection of adaptive time window further improves the contrast of photoacoustic imaging to 9.57. Our method effectively improves the contrast of photoacoustic imaging through scattering media.
